# An Updated Review Summarizing the Anticancer Efficacy of Melittin from Bee Venom in Several Models of Human Cancers

**DOI:** 10.3390/nu15143111

**Published:** 2023-07-12

**Authors:** Pratibha Pandey, Fahad Khan, Minhaj Ahmad Khan, Rajnish Kumar, Tarun Kumar Upadhyay

**Affiliations:** 1 Department of Biotechnology, Noida Institute of Engineering & Technology, Greater Noida 201306, Uttar Pradesh, India; shukla.pratibha1985@gmail.com; 2 Department of Biochemistry, School of Bioengineering and Biosciences, Lovely Professional University, Phagwara 144411, Punjab, India; minhaj.15324@lpu.co.in; 3 Department of Pharmaceutical Chemistry, Noida Institute of Engineering and Technology (Pharmacy Institute), Greater Noida 201306, Uttar Pradesh, India; rajnishkumar.pharmacy@niet.co.in; 4 Department of Biotechnology, Parul Institute of Applied Sciences and Centre of Research for Development, Parul University, Vadodara 391760, Gujarat, India; tarun_bioinfo@yahoo.co.in

**Keywords:** apitherapy, cancer therapeutics, honeybee venom, nanotechnology, melittin

## Abstract

Apitherapy (using bee products) has gained broad recognition in cancer therapeutics globally. Honeybee venom has a broad range of biological potential, and its utilization is rapidly emerging in apitherapy. Bee products have significant potential to strengthen the immune system and improve human health. Thus, this review is targeted toward recapitulating the chemo-preventive potential of melittin (MEL), which constitutes a substantial portion of honeybee venom. Honeybee venom (apitoxin) is produced in the venom gland of the honeybee abdomen, and adult bees utilize it as a primary colony defense mechanism. Apitoxin comprises numerous biologically active compounds, including peptides, enzymes, amines, amino acids, phospholipids, minerals, carbohydrates, and volatile components. We are mainly focused on exploring the potential of melittin (a peptide component) of bee venom that has shown promising potential in the treatment of several human cancers, including breast, stomach, lung, prostate, ovary, kidney, colon, gastric, esophageal, cervical cancers, melanoma, osteosarcoma, and hepatocellular carcinoma. This review has summarized all potential studies related to the anticancerous efficacy of melittin (apitoxin), its formulations, conjugates, and nano-formulations against several human carcinomas, which would further pave the way for future researchers in developing potent drugs for cancer management.

## 1. Introduction

Cancer morbidity and mortality rates are so high around the globe that it is vital to find effective management strategies [1]. Surgery, radiation therapy, chemotherapy, gene therapy, and hormone therapy make up most of the available therapeutic options today. These conventional medicine techniques are usually linked to severe unanticipated consequences that make management difficult [2]. There has been a great rush to develop alternative treatment strategies to avoid the typical adverse effects of conventional medications. The use of biotoxins as cancer treatment agents, such as animal venom, has also gained popularity [3]. These biotoxins are known to have toxicological and pharmacological effects and are created by living things as a defense against predators [4].

Apitherapy, or using bee products, is a complementary approach to some diseases’ treatment that aims to protect and enhance health, boost the immune system, and use bee products as a nutrient. Studies on bee venom have started to pick up in recent years, even though bee products, like honey, propolis, and pollen, have been known and utilized for a long time. Due to their rich content, these products have been used as food for many years and as a source of healing, from mending wounds to having an anticancer impact. The popularity of apitherapy products has grown even further due to scientific research [5]. In the animal realm, bee venom is a unique weapon. The primary defense function of the bee venom system is to protect the bee colony. It is a potent and sophisticated combination of chemicals to defend bees from predators, including vertebrates and other anthropophagi. Adolapin, melittin, apamin, enzymes, mast cell degranulating peptides, and non-peptide components, including histamine, dopamine, and norepinephrine, are physiologically active peptides found in bee venom from the venom gland found in the abdominal cavity [6].

Bee venom, often called apitoxin, is a complicated substance secreted by the bee venom gland, located in the abdominal cavity of bees, and injected into victims using a stinger. It may lead to localized inflammation and start a body’s immunological reaction [7]. The primary components of bee venom are amphipathic polycationic peptides, like amines and melittins, enzymes (phosphatase A2), and low-molecular-weight substances, such as active amines. Bee venom has been used for immunotherapy, Parkinson’s disease treatment, acupuncture, and apitherapy, which involves injecting the patient with an analgesic and anti-inflammatory drug. Furthermore, bee venom has numerous potential anticancer uses, including antimutagenic and radioprotective properties. Bee venom has also been used in other human diseases, such as multiple sclerosis and rheumatoid arthritis [8]. Table 1 summarizes all the potent bee venom phytocompounds reported in numerous cancers.

In several research studies, the melittin component of bee venom has shown significant inhibition of cell proliferation of several human cancer cells via stimulation of lymph nodes associated with local cellular immune response. The mechanism behind this bee venom-mediated growth inhibition includes apoptosis, lysis, and necrosis. Additionally, melittin also possesses antimutagenic, anti-inflammatory, radioprotective, and anti-nociceptive activities [9]. Further sections will decipher the studies related to the anticancerous potential of melittin, analogs of melittin, and formulations and nano-formulations of the melittin component of bee venom.

## 2. Melittin and Its Analogs

**Figure 1:**
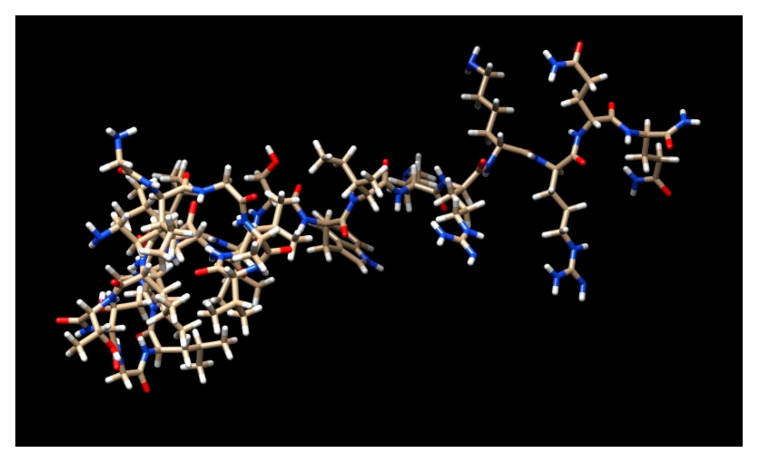
3D structure of melittin.

Melittin, which makes up around 50% of the weight of dried bee venom, is the main component of bee (*Apis mellifera*) venom. The linear structure of this amphiphilic peptide contains 26 amino acids. Melittin has comprehensive anti-inflammatory properties when used in modest amounts, but it can cause itchiness, inflammation, and localized pain when used in large doses. About 52% of the dry mass of bee venom is made up of melittin, a significant component (Figure 1) [10]. Numerous research studies have examined the impact of melittin’s regulation of apoptosis and the factors that cause apoptosis in different cancer types, including breast, ovarian, prostate, and lung cancer. Its molecular weight is 2840 Da, and it has 26 amino polypeptides in it [10].

Prepromelittin, an inactive precursor with 70 amino acids, is created in the venom gland of a bee during the process of making this peptide. Prepromelittin activation is a multistep procedure. An exoprotease removes a 22-amino acid fragment with proline or alanine at even-numbered positions after first being broken down into a predominantly hydrophobic 21-amino signal peptide, followed by the formation of a carboxy-terminal amide and the loss of the terminal glycine. The first 20 amino acids (starting at the *N*-terminus) of melittin’s active form are hydrophobic, whereas the next 6 are hydrophilic, giving it polar characteristics. At physiological pH, the *N*-terminal region has +4 charges, while the *C* terminal has +2 charges, totaling +6 charges. Melittin spontaneously forms a monomeric alpha-helix when attached to the cell membrane’s lipid bilayer under normal physiological circumstances. Melittin typically includes a random coil. A proline residue-containing coil connects the two helices that comprise the melittin monomer. As a result, this peptide can pass through the cell membrane and interact with cellular substructures at the molecular level [11].

TT-1 (Melittin’s mutant) was created by shortening the peptide chain and swapping glycine residues for lysine residues. The original peptide sequence was modified to “KIK AVL KVL TT,” which only had 11 amino acids, from “GIG AVL KVL TTG LPA LIS WIK RKR QQ”. In contrast to melittin, the TT-1 mutant exhibits improved stability and lesser toxicity due to its retention of the amino-terminal active site region. It also has increased hydrophobicity and a lowered net charge. According to a study, TT-1 inhibited the growth of TT cells by causing apoptosis by upregulating Bax, downregulating B cell lymphoma-2 (Bcl-2), and activating caspase-3 and -9 at the transcriptional and translational levels, respectively. These results demonstrated the therapeutic potential of TT-1 against thyroid cancer [12].

Chemotherapy resistance is a significant barrier to treating HCC patients. Another analog of melittin, MEL-pep, has shown significant anticancerous potential in human 5-fluorouracil-resistant hepatocellular carcinoma cells (BEL-7402/5-FU), and its treatment ruptures cell membranes and reverses 5-fluorouracil resistance. MEL-pep reduced P-glycoprotein expression, which is essential for the emergence of drug resistance to anticancer medications, by decreasing the PI3K/Akt signaling pathway. Intratumoral treatment of MEL-pep suppressed tumor growth in a dose-dependent manner in a mouse xenograft tumor model derived from BEL-7402/5-FU cells. Therefore, MEL-pep could be an excellent therapeutic approach for treating HCC resistant to chemotherapy [13].

Due to their effectiveness and high specificity, immunotoxins with selective cytotoxicity are often employed in solid-organ transplantation as therapeutic immunosuppressive drugs. This study describes a novel recombinant immunotoxin called anti-CTLA-4-scFv-melittin made from Escherichia coli and intended to eliminate activated T cells while preventing a general loss in systemic immunity. This fusion protein has the characteristics of minimal immunogenicity and selective cytotoxicity to CTLA-4 +ve T cells. It comprises an anti-CTLA-4-scFv unit and a melittin analog unit [14].

## 3. Molecular Interaction of Melittin (Honeybee Venom) in Cancer

**Figure 2:**
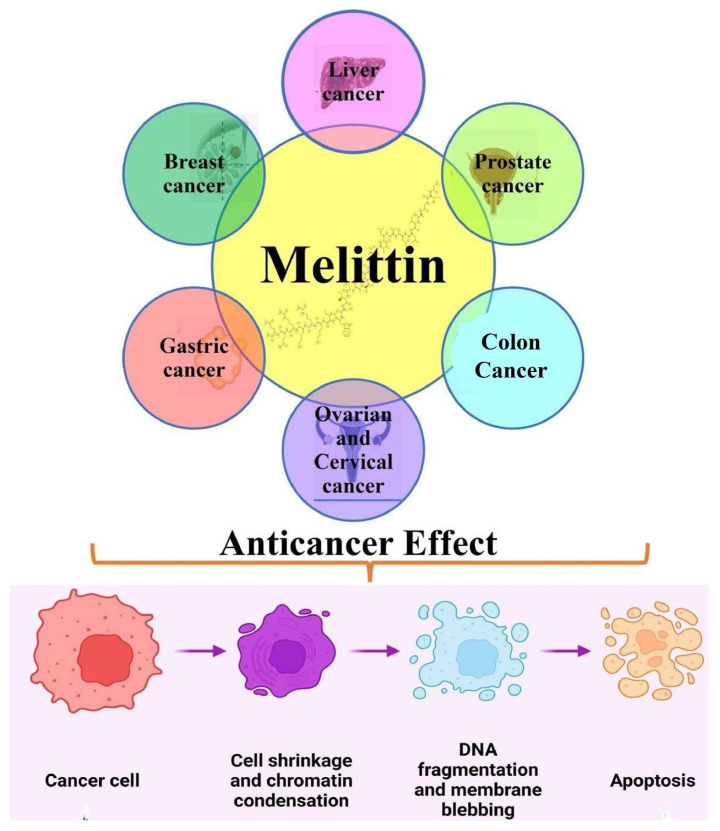
Anticancer potential of melittin in various preclinical cancer models by promoting chromatin condensation, DNA fragmentation, and causing cancer cell death.

Genes that code for proteins that can be essential players in intracellular signaling pathways frequently become mutated in cancer, and both genetic and environmental factors can influence these changes. Through anticancer chemicals, like bee venom and its primary component, melittin, cancer cells can be eliminated by interfering with the cell cycle and apoptotic cell death. In numerous studies, melittin has been shown to have an anticancer activity by causing apoptotic cell death in cancer cells, including those from the prostate, ovary, melanoma, lung, breast, bladder, leukemia, and hepatocellular carcinoma [15,16,17,18] (Figure 2).

**Figure 3:**
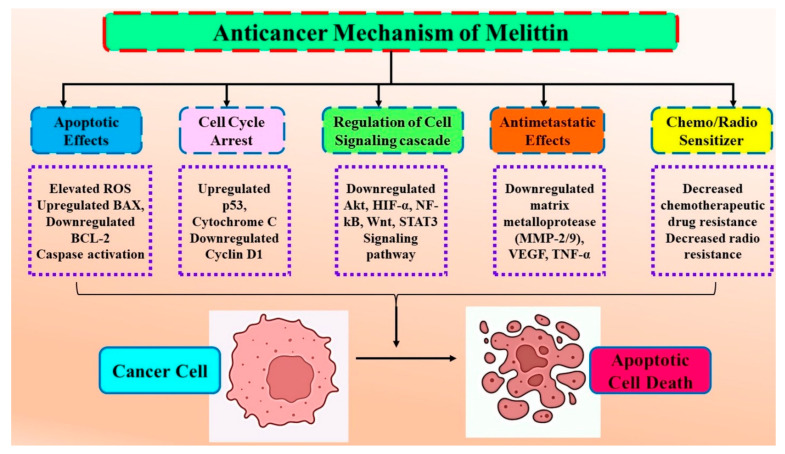
Anticancer mode of action of melittin on the essential molecular targets in different carcinomas. Melittin exerts its anticancer effects by inducing apoptosis, cell cycle arrest, modulation of the oncogenic signaling pathway, inhibiting metastasis, and promoting chemo/radio sensitivity. The vital molecular targets associated with melittin’s growth inhibitory and apoptotic inducing potential are Bax, Bcl-2, caspases, Akt, HIF-α, NF-kB, Wnt, STAT3, MMPs, VEGF, and TNF-α.

Bee venom has also been recognized as a traditional Chinese medicine for treating numerous malignancies, such as rheumatoid arthritis. Melittin plays a significant role, including apoptosis induction in several human cancer cells [19,20,21,22,23]. Melittin treatment induces apoptosis in human gastric cancer (SGC-7901) cells via modulating mitochondria pathways. These alterations increased apoptotic influencing factors, endonuclease G, and cytochrome C release, accompanied by caspase-3 activation and apoptosis in human GC cells [24]. Melittin-stimulated SGC-7901 cells displayed distinctive apoptotic morphology, significant reactive oxygen species (ROS) generation, and reduced MM potential. Table 2 summarizes the anticancerous potential of melittin in numerous human cancers (Figure 3).

### 3.1. Anticancer Efficacy of Melittin in Liver Cancer

Melittin (a water-soluble toxic peptide) from bee venom of Apis mellifera exhibited significant growth-inhibiting effects, including motility and viability, in hepatocellular cancer. Both in vitro and in vivo findings have shown suppressed Rac1-dependent efficacies, microfilament depolymerization, and cell motility, thereby suggesting melittin as an excellent therapeutic candidate for HCC via suppression of the Rac1-dependent pathway [26]. Later, cytotoxicity of apamin and melittin was reported in human hepatic (L02 and HepG2) cells. It was found that both the components of bee venom displayed growth arrest and initiation of early apoptosis in treated HepG2 cells in both time- and dose-dependent ways [26].

Melittin treatment significantly reduced tumor development, VM formation, and tumor HIF-1 expression in the xenograft tumor model in nude mice. Melittin also dramatically reduced the migration, invasion, and vimentin production of liver cancer cells caused by CoCl_2_. Treatment with CoCl_2_ increased the expression of N-cadherin and vimentin while suppressing E-cadherin. These EMT indicators experienced alterations at both the protein and mRNA levels, which melittin reversed. A CoCl_2_-induced buildup of HIF-1 enhanced the production of VEGF and MMP-2/9 and increased the level of phosphorylated Akt. Melittin reduced HIF-1 levels, repressing p-Akt, VEGF, and MMP-2/9 levels. This study concludes that melittin may prevent hypoxia-induced VM development and EMT in liver cancer by blocking the HIF-1/Akt pathway [27].

Melittin synergistically potentiated anticancer effect of sorafenib against liver cancer (HepG2) cells. Melittin treatment resulted in upregulated expression of p53, Cas7, Bax, PTEN, and Cas3, and downregulated expression of Bcl-2, Rac1, HIF-1a, Cyclin-D1, MMP9, Nf-κB, and VEGF. Growth arrest (at the G2/M phase) was reported in melittin-treated HepG2 cells, with significant changes reported in oxidative stress marker levels, such as MDA, GPx, SOD, and CAT [28]. Altogether, these findings strongly indicated the anticancerous potential of melittin in hepatocellular cancer treatment.

### 3.2. Anticancer Efficacy of Melittin in Breast Cancer

Activated EGFR signaling pathways have been reported to be positively associated with invasion and metastasis in MDA-MB-231 cells. EGF stimulation has been shown to promote invasive activities in the melittin treated MDA-MB-231 cells. Melittin treatment inhibited EGF-induced charge and MMP-9 expression via blocking NF-κB and PI3K/Akt/mTOR signaling pathways in breast cancer cells [29]. Another research study reported the better anticancerous potential of melittin compared to cisplatin and doxorubicin on 4T1 cell lines via upregulating the mRNA expression of Drp1 and Mfn1 genes [30]. Duffy et al., 2020, further unveiled the mechanism behind the anticancerous potential of melittin via suppressing the activation of HER2 and EGFR expression in breast cancer cells. Melittin administration enhanced docetaxel efficacy in suppressing breast tumor growth in an allograft model [31]. HIF-1α plays a vital role in the formation of the TME (tumor microenvironment) via the regulatory gene of anaerobic respiration and angiogenesis. Melittin inhibited the expression of genes involved in tumor microenvironment (TME) formation via disruption of HIF-1α signaling in breast cancer (MDA-MB-231) cells [32]. Another study investigated the effect of melittin treatment on tumor growth in the 4T1 xenograft breast cancer mouse model. Radiosensitivity was assessed in melittin-treated cells by measuring growth inhibitory potential in altered tumor volume. Melittin displayed significant growth inhibition in 4T1 and MCF-7 breast cancer cells, reducing clonogenicity. Combinatorial treatment of melittin and irradiation potentially enhanced the Bax/Bcl-2 ratio. In vivo, intraperitoneal injection of melittin significantly reduced tumor growth in 4T1 tumor-bearing mice. Thus, melittin emerged as a promising radiosensitizer for breast cancer radiotherapy via inducing apoptosis [33].

### 3.3. Anticancer Efficacy of Melittin in Gastrointestinal Cancers

Mahmoodzadeh et al. (2015) isolated melittin using reversed-phase HPLC and assessed its efficacy against AGS cells. Melittin-treated AGS cells displayed inhibited cell growth (in a dose/time-dependent manner). Different molecular assays exhibited significant necrosis in melittin-treated AGS cells [34]. Melittin treatment (<1 µM), mediated cytotoxicity via necrosis in gastrointestinal epithelial cells [35].

Further, melittin treatment induced apoptosis in SGC-7901 (human gastric cancer) cells via an activated mitochondrial pathway. Melittin-treated cells SGC-7901 displayed significant apoptosis induction with altered ROS levels, apoptotic morphologies, reduced mitochondrial membrane permeability, and caspase-3 activation [24]. Melittin demonstrated antitumor potential against numerous human gastrointestinal cancers. Another study indicated reduced cell viability, colony formation, and cell adhesion. Furthermore, melittin decreased cell motility and suppressed the invasion and migration of AGS cells. It further reduced protein expression of the MMP-2 and Wnt/BMP signaling pathways [36].

Nikodijević et al. further displayed the anticancer potential of melittin on colorectal cancer cells (HCT-116/SW-480) and induced proapoptotic activity via regulating apoptosis signaling proteins (Fas receptors, Bcl-2, and caspase 9) [19]. Another study evaluated the cytotoxic efficacy of melittin on HCT116 (human colon carcinoma) cells and the synergistic potential of MEL and PLA2 on HCT116 cells. Melittin and PLA2-treated HCT116 cells showed significant cytotoxicity effects and membrane disruption, thereby validating Ntheir cytotoxic effects [37]. In colorectal cancer cells, melittin treatment inhibited cell viability and induced apoptosis in SW480 cells via modulating apoptosis-associated proteins. It triggered ER (endoplasmic reticulum) stress, resulting in unstable calcium homeostasis and apoptosis in SW480 cells. In SW480 tumor-bearing mice, melittin treatment reduced tumor growth [38]. Cell viability assay, colony formation assay, and electro-mobility shift assay displayed NF-κB DNA binding activity in colon cancer. Melittin inhibited colon cancer cell growth via apoptosis induction and increased the expression of death receptors (4 and 5), p21, p53, and caspases (3, 8 and 9). The treatment further reduced NF-κB DNA binding activity, which validated its growth-inhibitory potential in colon cancer cells [39].

### 3.4. Anticancer Efficacy of Melittin in Gynecological Cancers

Melittin efficacy was further evaluated against gynecological cancers. Melittin inhibited cell growth via enhancement of DT (death receptor) expression, increased caspase and Bax activation, apoptosis induction, and STAT3 pathway inhibition in PA-1 and SKOV3 (human ovarian cancer) cells [40]. Fluvastatin and melittin treatment showed enhanced antineoplastic activity and improved cytotoxic potential in OVCAR3 (human ovarian cancer) cells. FLV-MEL displayed growth arrest in G2/M and pre-G1 phases and limited cells in S and G0/G1 cell cycle phases [41].

Melittin from Iranian honey bee venom (using reversed-phase HPLC) displayed anticancer potential on human cervical cancer cells via apoptosis induction. Melittin inhibited cell proliferation (by MTT assay) and induced apoptosis in cervical cancer cells. These results suggest that melittin induces apoptotic cell death (via flow cytometric assay) in cervical cancerous cells [42,43].

### 3.5. Anticancer Efficacy of Melittin in Other Human Cancers (Hepatocellular; HNSCC; Lung)

In vitro studies reported that melittin inhibited cell proliferation and potentially reduced CyclinD1 and CDK4 expressions in HepG2 cells. Western blot and RT-PCR analysis reported upregulated HDAC2 and PTEN expressions in melittin-treated HepG2 cells. Additionally, melittin treatment reported downregulated Akt phosphorylation, which indicated the inhibitory potential of melittin in hepatocellular carcinoma via the inhibition of the PI3K/Akt cell signaling pathway [46].

Another study evaluated the probability of melittin on the radio sensitization of hypoxic HNSCC (head and neck squamous cell carcinoma). Melittin treatment inhibited cell growth and cell apoptosis and reduced expression levels of VEGF and HIF-1α proteins correlated with hypoxia cell radioresistance. Furthermore, administration of melittin via intraperitoneal injection in CNE-2 tumor-bearing mice reduced the HNSCC tumor growth. Thus, melittin appeared to be a potent radiotherapy sensitization agent with its remarkable anti-hypoxia activity [47]. Tumor-associated macrophages have an M2-like phenotype, facilitating tumor progression via increasing immunosuppression and angiogenesis. In mouse models bearing lung cancer, melittin inhibited tumor growth and increased TAMs M1/M2 ratio by selectively decreasing CD206+ M2-like TAMs, VEGF, CD31 (angiogenesis marker), and Mrc1/CD206 protein and gene expression in tumor cells. Altogether, this study validated the therapeutic efficacy of melittin in tumor treatment via targeting M2-like TAMs [48].

Wang et al. investigated the anticancerous efficacy of melittin in PDCA models (model of pancreatic cancer). Melittin suppressed tumor growth by inducing cell-cycle growth arrest and apoptosis in PDCA cells. Microarray analyses further displayed downregulation of the clusterin gene of cholesterol biosynthesis, which further boosted gemcitabine sensitivity by inhibiting CLU expression in PDAC cells. Furthermore, combined treatment of gemcitabine and melittin in a xenograft mouse model displayed more efficient inhibitory potential against PDCA tumor growth [49]. Antitumor effects of melittin were assessed in NSCLC (non-small cell lung cancer) cells and exhibited the inhibition of EGF-induced migration and invasion. Subcutaneous administration of melittin suppressed NSCLC tumor growth and reduced VEGF and HIF1-α protein expression [50]. Melittin displayed significant cytotoxicity to ChaGo-K1 (human bronchogenic cancer cells) via inducing cell cycle arrest (G1 phase) and apoptosis. Melittin, therefore, can be utilized as an alternative therapeutic agent for managing lung cancer due to its cytotoxicity against ChaGo-K1 cells and blocking the THP-1 cell differentiation into TAMs [51]. Melittin exhibited cell death with significant cellular changes and damaged membranes in colorectal and gastric cancer cell lines (AGS, HCT-15, and COLO205) [52].

MEL-dKLA induced discerning cell death of M2 macrophages without affecting other leukocytes (dendritic and T cells). These alterations resulted in reduced tumor weight and angiogenesis. Intriguingly, although MEL and MEL-dKLA decreased the proportion of CD206+ M2-like TAMs in tumors, only MEL-dKLA caused apoptosis in CD206+ M2-like TAMs; MEL did not cause cell death. Our research showed that MEL-dKLA could be a viable cancer treatment drug by targeting M2-like TAMs [53]. Yu et al.’s study showed that melittin inhibited NSCLC cells migration, invasion, and proliferation while inducing cell death. Caspase-3 and Apaf-1 gene expression were both induced by melittin. In NSCLC cells, melittin decreased the expression of tumor growth factor TGF-β and phosphorylated total ERK (pERK/tERK). TGF-overexpression (pTGF) in non-small cell lung cancer (NSCLC) cells eliminated melittin-reduced TGF-β-expression and pERK/tERK. Compared to the control group, melittin treatment raised TUNEL-positive compartments and lowered TGF-β- and ERK expression levels in tumor tissue. Melittin may be a promising anticancer agent for the treatment of NSCLC, according to the study’s findings, which showed that it inhibited the proliferation, migration, and invasion of NSCLC cells and caused apoptosis via down-regulating the TGF-β-mediated ERK signaling pathway [54].

## 4. Conjugates of Melittin and Its Anticancer Potential

In some studies, melittin treatment did show some limitations, including toxicity, non-specificity, degradation, ineffective systemic transport, low bioavailability, and hemolysis in cancer cells [55]. Thus, multiple strategies have been used to combat the problem of using melittin for cancer therapy. To improve melittin’s efficacy, selectivity, and specificity in cancer therapy in vitro and animal model systems, researchers are actively using nanotechnology, formulations, gene therapy, and immunoconjugates. Table 3 summarizes all possible formulations of melittin developed to enhance the anticancerous potential of melittin. Cytokine fusion protein holds great potential for cancer immunotherapy via a modulating immune response. IL-2 is a highly effective therapeutic option for advanced cancers, but the therapeutic efficacy of this cytokine signaling component has limitations of severe systemic toxicity. Melittin emerges as an attractive anticancer agent with its wide range of lytic potential. The melittin-MIL-2 was more effective in inducing T cell and NK cell cytotoxicity. In tumor tissues, melittin-MIL-2 (bifunctional fusion protein: melittin and mutant IL-2) treatment promoted IFN-γ secretion and reduced immunosuppressive cells. Melittin-MIL-2 can elicit strong antitumor potential and immune stimulation, and could be considered a potent candidate for cancer immunotherapy [56]. The biosynthesized nanoscale peptide melittin damaged microbial phospholipid membranes by creating stable or momentary pores. A mouse model of a human HCC xenograft tumor was created using a non-viral vector (pSURV-Mel), which encodes the Mel gene. In tumor cells, the survivin promoter is preferentially activated. The pSURV-Mel plasmid selectively produced Mel in tumor cells and also promoted cytotoxicity.

Additionally, intra-tumoral injection of pSURV-Mel substantially inhibited the development of xenograft tumors. An apoptosis-dependent mechanism was used by pSURV-Mel to cause cell death [57]. A novel dual-secured architecture allowed us to inhibit the hemolytic activity of melittin in DSNS. These experiments showed that a zwitterionic polymer and redox-sensitive linkages provide a novel method for administering therapeutic peptides that is both secure and efficient [58].

Methyl-CpG binding protein 2 (MeCP2) is vital for tumor growth, apoptosis, migration, and invasion. In SMMC-7721 cells and human HCC tissues, MeCP2 was highly expressed. In SMMC-7721 cells, MeCP2 overexpression stimulated cell growth, while MeCP2 silencing decreased cell proliferation. Melittin decreased MeCP2 expression and caused cell growth to arrest at the G0/G1 phase of the cell cycle, which prevented cell proliferation. Furthermore, melittin administration markedly reduced the expression of Shh and GLI1. Melittin reduces MeCP2 through Shh signaling in SMMC-7721 cells to decrease cell growth [59].

The cytokine growth-dependent cell line CTLL-2 was used to investigate the IL-2 activity of the melittin-MIL-2 fusion protein. Melittin-MIL-2 was more efficient in triggering T cells’ and NK cells’ cytotoxicity. Melittin-MIL-2 therapy reduced immunosuppressive cells and increased IFN-γ secretion in tumor tissues. In addition, the fusion protein decreased breast cancer lung metastasis. According to this research, melittin-MIL-2 is a viable option for cancer immunotherapy since it can enhance immune activation and have anticancer effects [56].

PEI (polyethylenimine) was targeted with chlorotoxin (CTX) via N-succinimidyl 3-(2-pyridyldithio) propionate (SPDP) cross-linker. CTX can bind specifically to matrix metalloproteinase-2, which is overexpressed in numerous carcinomas. Flow cytometry analysis revealed that targeted nanoparticles’ transfection efficiency is significantly higher than non-targeted nanoparticles. Targeted nanoparticles carrying the melittin gene also decreased the cell viability of PC3 cells significantly, while no toxic effects were observed on the NIH3T3 cell line. Therefore, CTX-targeted nanoparticles carrying the melittin gene could be an appropriate gene delivery system for prostate and other MMP-2-positive cancer cells [60]. The crucial stages in determining the aggressive phenotype of human malignancies are metastasis and invasion of the primary tumor. In macrophage cells, melittin blocks cyclophilin A (CypA), a widely present peptidylprolyl cis–trans isomerase. The results of the Transwell experiment in the current study indicated that melittin might, in a dose-dependent manner, downregulate the invasion level of MCF7 cells. Flow cytometry and reverse transcription polymerase chain reaction also discovered that melittin lowered the expression of clusters of differentiation (CD)147 and matrix metallopeptidase 9 (MMP9), whereas CypA elevated CD147 and MMP9 expression. Overall, the results of the present investigation suggested that melittin reduced the level of invasion of MCF-7 cells by downregulating CD147 and MMP9 and reducing CypA production [61].

To develop a successful co-delivery system for cancer therapy, the anticancer drugs doxorubicin (DOX) and melittin (MEL) were electrostatically loaded onto the surface of CA-MNPs. A coprecipitation technique was used to create the citric acid-functionalized Fe3O4 magnetic nanoparticles (CA-MNPs), which were then thoroughly characterized. MTT assay revealed a synergistic interaction between DOX and MEL in MCF-7 breast cancer cells, resulting in noticeably increased antitumor activity compared to solo administration of both anticancer drugs at equal doses. According to the findings, the co-delivery system of CA-MNPs loaded with (DOX/MEL) is particularly suitable for application in magnetically targeted cancer therapy [62].

Numerous reports have demonstrated lytic and apoptotic effects of melittin in several malignant cell lines. Antinucleolin aptamer (AS1411) was covalently joined to melittin. Combinatorial treatment of the aptamer–melittin demonstrated effective cell uptake and was more cytotoxic in A549 cells than melittin alone. When compared to free melittin, aptamer–melittin showed considerably less hemolytic activity. This study demonstrated that melittin might be delivered precisely to A549 cells by being covalently coupled to an anti-nucleolin aptamer (AS1411) which can further lessen the cytotoxic efficacies of melittin on cells without nucleolin receptor expression [63]. Through oxidative stress-mediated pathways, plasma-treated phosphate buffered saline solution (PT-PBS), a resolution high in reactive oxygen and nitrogen species (RONS) can impair the integrity of cell membranes and cause cancer cell death. PT-PBS was, therefore, combined with MEL to increase its penetration into cancer cells and decrease the necessary therapeutic dose. The results collectively point to MEL and PT-PBS as potentially effective combination therapies to avoid the non-specific toxicity of MEL, which may aid in clinical applicability in the future [64].

The melittin-loaded noisome down-regulated the expression level of MMP9, Bcl2, and MMP2 genes while upregulating the expression levels of Caspase 9, Bax, and Caspase 3 genes. They inhibited cell migration and enhanced apoptosis in both the cell lines compared to melittin samples. Histopathology findings also revealed a reduced mitosis index, pleomorphism, and invasion in melittin-loaded dangerous cells [65]. Melittin induced apoptosis via disrupted MMP, the accompanying release of ROS, and upregulated expression of caspases (-3 and -9), Bax, and poly (ADP-ribose) polymerase 1 in pcTERT–melittin transfected TE1 cells. pcTERT–melittin treated TE1 cells showed significant growth arrest in the G0/G1 phase followed by reduced cyclin D1, CDK4, and CDK6 expression levels. These findings demonstrated that pcTERT–melittin induced apoptosis and inhibited tumor metastasis in esophageal cancer cells [66]. In a study, Mel-Lips (melittin liposomes) and Mel-HA-Lip (hyaluronic acid-modified Mel-Lip) reduced the toxicity and increased the antitumor efficacies of melittin. HA modification increased liposomal internalization and resulted in stronger sustained release effects within the treated cells compared to Mel-Lip treated cells [67].

Combined treatment of 5-FU and MEL (5-fluorouracil and melittin) displayed enhanced cytotoxic efficacies against A431 cells (skin squamous cell cancer). MEL-induced plasma membrane disintegration leads to increased sensitization of cells to 5-FU. These treatments resulted in apoptosis via increased DNA fragmentation, disrupted mitochondrial metabolism, and growth arrest in the S and G2/M phases of the cell cycle. 5-FU+MEL represented an innovative strategy for the management of skin squamous cell cancer [68]. Furthermore, the study investigated the growth inhibiting potential of melittin–dKLA against M2 macrophages and displayed significant growth inhibition and apoptosis of M2-like macrophages. Melittin–dKLA significantly inhibited the proliferation and migration of M2 macrophages, resulting in reduced tumor growth [69].

Melittin (MEL) is an antineoplastic agent that has shown promise as a treatment for cancer patients. Compared to the basic fluvastatin (FLV), the combination of FLV PL ALA MEL (fluvastatin with melittin loaded with a combination of phospholipid and alpha lipoic acid) showed enhanced cytotoxic potentiality. A higher number of cells accumulated over the G2/M and pre-G1 phases. In contrast, the G0/G1/S phases saw the accumulation of fewer cells, according to studies analyzing cell cycle, wherein optimized FLV PL ALA MEL nanoparticles were found to inhibit Caco2 colon cancer cells more significantly than other therapeutic treatments. An innovative and more effective treatment for colon cancer may be made possible by the optimized formulation [70].

Adding Dap^AMCA^ (Trp19 substitution with non-canonical fluorescent AA) residue to melittin altered its mechanism of action with the cell membrane, resulting in reduced hemolytic toxicity and an increased selectivity index, with an up to a five-fold increase in comparison to melittin, according to in vitro hemolytic and anticancer activity studies. MEL^FL^ (Dap^AMCA^-labeled melittin) in cancer cells has shown significant nuclear and nucleolar localization capabilities and high membrane penetration [71].

**Table 3:** Summarized anticancerous potential of melittin formulations in cancer.

MEL Conjugates	In Vitro/In Vivo	Modes of Action	References
Melittin–MIL-2 fusion protein	SKOV3 cells	Growth inhibition Inducing NK cell and T cell cytotoxicity Increased IFN-γ production	[56]
Non-viral vector (pSURV–Mel)	HCC cell lines A mouse model with a human HCC xenograft tumor	Induced cytotoxicity Suppressed growth of xenograft tumors	[57]
Dual secured nano-melittin	MCF-7 cells HCT-116 cells SKOV-3 cells NCI/ADR-RES cells	Increased toxicity Apoptosis induction	[58]
Ad-rAFP–Mel (Recombinant adenoviruses carrying the Mel gene and α-fetoprotein)	BEL-7402 cells Nude mice carrying transplanted tumor	Reduced tumorigenicity rates Antineoplastic effect	[72]
Melittin/Avidin conjugate	DU 145 cells SK-OV-3 cells	Strong cytolytic activity High MMP2 activity	[73]
Ad-rAFP–Mel	BEL-7042 cells	Apoptosis induction	[74]
Immunoconjugates having melittin-like peptide 101	Mouse MAbs, J591 and BLCA-38	Xenograft tropism Delay in tumor growth Increased mouse survival	[75]
Ad-rAFP–Mel	Bel-7402	Apoptosis induction Induced Fas expression	[76]
Melittin into perfluorocarbon nanoparticle	Murine tumors	Dramatic reduction in tumor growth Trigger apoptosis	[77]
Immunoliposomes having trastuzumab and melittin	SKBr3 cells	Reduced cancer cells viability Reduced HER2 expression	[78]
α-melittin-NP	Melanoma-bearing mice	Inhibited growth of melanoma cells Increased cytotoxicity for melanoma cells	[79]
AM-2 (peptide)+melittin	HepG2 cells	Increased melittin affinity Increased binding and apoptosis efficacy	[80]
sTRAIL–melittin	K562 cells HepG2 cells	Increased cytotoxic and apoptotic potential	[81]
Melittin–MhIL-2 fusion protein	SKOV3 cells Ovarian cancer mice	Growth inhibition Inhibited tumor growth	[82]
M-IL-2(^88^Arg, ^125^Ala) fusion protein	SKOV3 cells	Growth inhibition Strong antigen-specificity	[83]
Mel–N (asparagine-substituted melittin) and Mel–S (serine-substituted melittin)	BV-2 cells	Inhibitory effects on IL-6 and TNF-α production	[84]
5-Fu + melittin	BGC-823	Synergistic effects on cytotoxicity of 5-FU Suppressed expressions of thy midylate synthetase, ERCC1 (excision repair cross-complementing gene 1), BRCA1 (breast cancer susceptibility gene 1), beta-tubulin III, and MAPT (microtubule-associated protein tau)	[85]
QG511-HA–melittin	Hep3B cells SMMC-7721 HCC xenograft	Strong inhibition effect on Hep3B and HepG2 cells Inhibited HCC xenograft growth	[86]
RhuPA1-43–melittin	SKOV3 cells	Growth inhibition Induced cell cycle arrest Induced apoptosis	[87]
rATF–mellitin	SKOV3 cells	Growth inhibition	[88]

## 5. Nanoformulations of Melittin and Its Anticancer Potential

**Figure 4:**
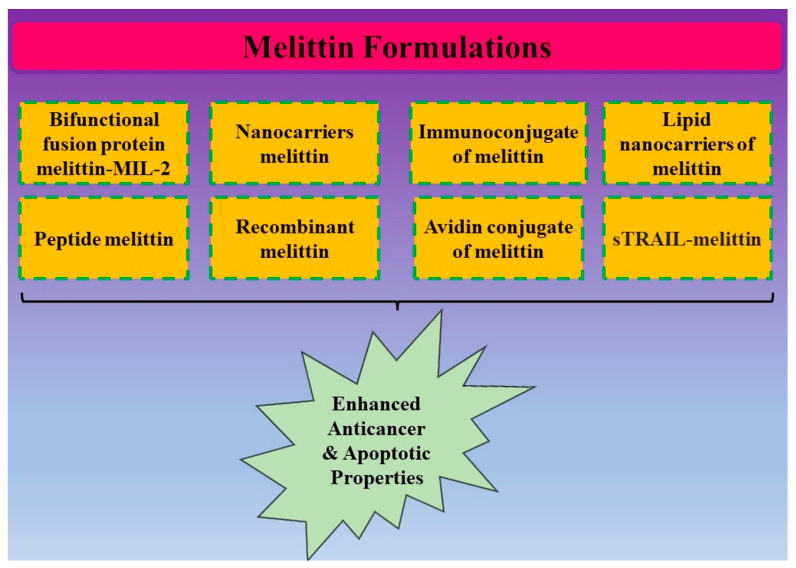
Different types of melittin formulations for enhanced anticancer- and apoptotic-inducing potential in cancer models.

Targeted cancer combination therapy has been proposed and verified using a unique genetically engineered vesicular antibody–melittin (VAM) drug delivery platform. It was possible for the bioactive and targetable nano-melittin conjugated by hGC33 scFv to be released at tumor locations in a way that was responsive to MMP14, which decreased off-target toxicity, especially the hemolytic activity of melittin. Importantly, VAM could be loaded with nanoparticles or small-molecule medicines for combination therapy. Nano-melittin disrupted phospholipid bilayers and created membrane gaps for the anticancer drugs co-delivered by VAM to reach deeper tumor locations and produce synergistic therapeutic effects. Specifically, the immunomodulatory impact of nano-melittin to activate the immune response was further increased by the punching effect produced by sonodynamic therapy [89,90]. Figure 4 summarized all possible conjugates and formulations of melittin synthesized for the enhanced drug delivery system.

Another study displayed enhanced antitumor effects of melittin when combined with biotoxins. Results from in vitro analysis showed that the chimeric gelonin–melittin fusion proteins maintained intrinsic activity in blocking protein translation that was equivalent to that of unmodified gelonin. However, recombinant chimeric gelonin–melittin fusion (rGel-Mel) and chemically conjugated gelonin–melittin (cGel-Mel) showed greater cell uptake, producing a significantly increased cytotoxic activity compared to treatment with gelonin, melittin, or a physical mixture of gelonin and melittin. Interestingly, gelonin’s IC50 was 32 and 10 times lower in the cell lines for cGel-Mel and rGel-Mel, respectively, than gelonin. Valency may be a key component in determining the degree of melittin-mediated cell uptake, as evidenced by the better antitumor activity of multivalent cGel-Mel to monovalent rGel-Mel [91].

Melittin nano-liposomes are more effective in treating hepatocellular carcinoma because of their potent antitumor effects and superior biological safety. According to preclinical and clinical investigations, the primary adverse effects were allergic response and pain at the administration site [92]. By encapsulating melittin with poloxamer 188 to reduce toxicity, melittin nano-liposomes were developed, and their inhibitory actions were investigated on liver cancer and biological safety. It was further demonstrated how melittin nano-liposomes drastically reduced the viability of hepatocellular carcinoma (HCC) cells in vitro and significantly slowed the development of subcutaneous and orthotopic HCC transplantation tumors in vivo. Compared to melittin, it caused less inflammation and allergy in mice [92].

A peptide vector of nanodiamonds (NDs) and PEGylated polyglutamic acid (ND@PLGPEG-co-PLGA) has been created. Electrostatic interaction was used to attach the positively charged peptide medicine melittin to the vector’s negative surface charges. In 2 days, almost no melittin was released from the nanoparticles under physiological pH conditions, indicating that the desorption of melittin from the surface was pH-dependent. In an acidic environment, however, a constant discharge was found. Based on the amount of MEL present in NDs, the bound melittin showed improved toxicity towards MCF-7 cells. According to these findings, when exposed to breast cancer cells, negatively charged polymer-coated NDs could release the cargo [93].

Membrane tests and flow cytometry tests showed that melittin complexed with nanographene oxide has a more toxic effect on breast cancer cells than melittin alone. In addition, cells can be shielded by nanodiamonds from melittin’s lytic effects. To increase its toxic effect, carbon nanoparticles, diamond, graphene oxide, and pristine graphene were utilized as melittin carriers on breast cancer cells (MDA-MB-231 and MCF-7). Compared to melittin, all complexes decreased but did not eliminate the level of necrosis. Results thus point to potential applications for melittin-carrying carbon nanoparticles in medicine [94].

Using calcium carbonate nanoparticles (CCN) as carriers and MUC1–Dimer aptamers as targeting agents, researchers were able to examine the impact of combining the anticancer drugs epirubicin (Epi) and melittin (Mel) on cancer cells. Epi was sensitive to pH when released from the MUC1–Dimer aptamer–CCN–Epi complex. Studies on cellular uptake revealed that the MUC1–Dimer aptamer–CCN–Epi complex internalized more readily into MCF-7 and C26 cells (target) than HepG2 cells (nontarget). Interestingly, the MUC1–Dimer aptamer–CCN–Mel and MUC1–Dimer aptamer–CCN–Epi complexes showed relatively reduced toxicity compared to target cells. Additionally, MCF-7 and C26 cells demonstrated significant synergistic cytotoxicity following the co-delivery of Epi and Mel using a combination of MUC1–Dimer aptamer–CCN–Mel and MUC1–Dimer aptamer–CCN–Epi complexes [16]. Its instability and quick breakdown significantly hampers practical therapeutic applications of MEL. The anticancer effects of PEG-GO-Fe3O4/MEL complexes on human cervical cancer HeLa cells were examined in the present work. Graphene oxide (GO)-based magnetic nanocomposites (PEG-GO-Fe3O4) were produced and used as the drug delivery vehicles of MEL. In vitro research showed that PEG-GO-Fe3O4/MEL significantly increased the inhibitory effect on HeLa cells and caused pore formation in the cell membrane, resulting in cell lysis. PEGylated GO is a MEL protector in this newly created drug delivery system, and Fe_3_O_4_ nanoparticles serve as magnetic responders. As a result, active MEL can be released over an extended period and sustain its inhibitory impact on HeLa cells [95].

A promising method for enhancing the effectiveness of cancer immunotherapy involves the targeted administration of a nano vaccine loaded with a tumor antigen and adjuvant to the lymph nodes. α-melittin nanoparticles significantly increase lymph node accumulation and APC activation compared to free melittin, which results in a 3.6-fold boost in antigen-specific CD8+ T cell responses. Furthermore, the growth of primary and distant tumors is significantly slowed by α-melittin nanoparticles in a bilateral flank B16F10 tumor model. As a result, α-melittin- nanoparticles act as efficient whole-cell lymph node-targeted nano vaccines by inducing a systemic antitumor response [96]. Melittin, which has been encapsulated, is still immunogenic and can be used as an adjuvant to trigger an immunological reaction that is lethal to the drug delivery carrier. With great safety and no loss of cytolytic capacity, D-melittin nanoformulations significantly reduce the immune response. D-melittin micelles (DMM) cause hemolysis and demonstrate substantial cytotoxicity in a pH-dependent manner. Additionally, DMM causes immunogenic cell death, highlighting its potential for cancer immunotherapy [97].

The PEG-modified MpG@LPN (melittin-loaded lipid-coated polymeric nanoparticle) displayed minimal hemolytic activity and nonspecific cytotoxicity even at high concentrations, according to assays for cell proliferation and hemolysis. Apoptosis induction and specific interaction with target tumor cells were made possible by the alteration of targeting molecules on the MpG@LPNs. As a result of in vivo tests, it was discovered that MpG@LPNs could effectively limit tumor growth without causing hemolysis or tissue damage. Results indicated that the developed MpG@LPN with a core–shell structure could successfully address the primary challenges faced by melittin in clinical applications and has significant promise as a cancer treatment [98]. AS1411 aptamers, melittin, and a gold nanoparticle were combined to create a new nano-complex for treating breast cancer cells. Tetrachloroauric acid reduction was used to generate gold nanoparticles (GNP). Melittin was modified with cysteine and bound to the gold nanoparticle using an AS1411 aptamer. This nano-complex was more cytotoxic in MCF-7 cells than in L929 cells, according to MTT data. These results made it abundantly evident that the gold nanoparticle-melittin-AS1411 combination could help deliver melittin specifically to cancer cells [99]. This review has outlined the anticancer potential of melittin as well as its formulations, conjugates, and nanoformulations, which have enhanced its efficacy and bioavailability, respectively. These developments could be used to further utilize the medicinal potential of melittin as a potent drug candidate for effective cancer management.

## 6. Conclusions

The accumulated evidence strongly suggests that melittin has the potential to be a cancer treatment. Melittin’s anticancer effects should be investigated in further detail and developed as an alternate method of cancer therapy. In many regions of the world where expensive chemotherapeutic medications are not available through the healthcare system, this strategy could be of great value if it is successful. Melittin has demonstrated substantial effectiveness in producing apoptosis, necrosis, mitochondrial disruption, prevention of angiogenesis, cell cycle arrest, and suppression of cancer cell invasion and metastasis. Melittin is a promising option for cancer treatment, but melittin’s in vivo lysis ability and some non-specific cytotoxicity have limited its use as a therapeutic agent in clinical settings. As a result, melittin in cancer therapy was improved by the use of nanotechnology techniques. The existing body of research strongly implies that melittin can be delivered using nanotechnology to target cancer cells, significantly increasing its therapeutic efficacy while having little or no hemolytic effect Altogether, it has further improved stability, specificity, efficacy and could further provide safer and cost effective lead candidate for cancer management. Melittin has a long way to go before it can be used clinically. Still, we firmly believe that continued research on the subject will eventually enable these molecules to be evaluated as a possible anticancer therapy in the future.

## Figures and Tables

**Table 1:** Components of honeybee venom.

Components of Bee Venom			
Melittin	Fructose	2-nonanol	Tertiapin
Apamin	Glucose	*n*-decyl acetate	Melittin F
MCD Peptide	Isopentanol	Phosphorous	Acid phosphatase
Secarpin	Benzyl acetate	Calcium	Hyaluronidase
Minimise	Isopentyl acetate	Magnesium	Phospholipase B
Procamine A, B	*n*-butyl acetate	Noradrenaline	Phospholipase A_2_
Protease inhibitor	*n*-octyl acetate	Histamine	a-Glucosidase
Cardiopep	Benzyl alcohol	Dopamine	Phospholipase

**Table 2:** Anticancerous potential of melittin in several models of human cancers.

Cancer	Cancer Model	Anticancer Effect	Molecular Target	Reference
Liver cancer	MHCC97L and MHCC97H cells; Nude mice	Antimetastatic, reduced cell migration, and motility	Rac1-dependent inhibition	[25]
	HepG2 cells	Cell growth arrest and apoptotic induction	-	[26]
	SMMC-7721, Huh7, and Hep G2 cells; xenograft tumor model	Tumor growth inhibition, reduced cell migration, and motility	Reduced expression of HIF-1α, VEGF, p-Akt, and MMP-2/9	[27]
	HepG2 cells	Cell growth inhibition and cell cycle arrest	Reduced expression of Bcl-2, VEGF, Nf-κB, HIF-1a, Cyclin-D1, Rac1, and MMP9 Increased expression of p53, Bax, PTEN, Cas7, and Cas3	[28]
Breast cancer	MDA-MB-231, MCF-7 cells	Reduced tumor cell migration and invasion	Inhibition of PI3K/mTOR/Akt/ pathway and NF-κB Reduced MMP-9 expression	[29]
	4T1 cells	Decreased cell proliferation and apoptotic induction	Upregulated Drp1 and Mfn1 mRNA expression levels	[30]
	MCF 10A, MCF-12A cells; allograft TNBC model	Apoptotic induction decreased chemoresistance	Suppressed HER2, MAPK, and EGFR activation	[31]
	MDA-MB-231 cells	Inhibited cell growth, apoptotic induction, and modulation of tumor microenvironment	Deregulated VEGFA, LDHA, and NFκB expression levels Increased TNFA and BAX expression levels Inhibition of HIF-1α cell signaling pathway	[32]
	4T1, MCF-7 cells; xenograft mouse model	Inhibited tumor growth, apoptotic induction, increased radiosensitivity and	Increased Bax Decreased Bcl-2	[33]
Gastric cancer	AGS cell line	Inhibited cell growth, and apoptotic induction	-	[34]
	SGC-7901 cells	Inhibited cell growth, and apoptotic induction	Enhanced ROS, caspase-3, cyt C, AIF, Endo G	[24,35]
	AGS cell line	Reduced cell viability, antimetastatic effect, reduced cell migration and motility	Reduced expression of Wnt/BMP and MMP-2 signaling pathway proteins	[36]
Colorectal cancer	HCT-116, SW-480 cells	Inhibited cell growth, apoptotic induction	Increased Fas receptors, caspase 9, and members of the Bcl-2 family	[19]
	HCT-116 cells	Increased cytotoxic effect, apoptotic induction	Membrane disruption	[37]
Colon cancer cells	SW480 cells; SW480 tumor-bearing mice	Suppressed cancer growth, apoptotic induction	Stimulated ER stress Imbalance in calcium homeostasis	[38]
	HT-29 cells	Antitumor and anti-inflammatory effects	Reduced expression of cyclooxygenase-2 (Cox-2), tumor necrosis factor-alpha (TNF-α), and interleukin one beta (IL-1β)	[39]
Ovarian cancer cells	SKOV3 and PA-1 cells	Inhibited cell growth and induced apoptosis	Increased expression of death receptors (DR3 and DR6) Increased expression of caspase-3, 8, and Bax Increased expression of cleaved caspase-3 Inhibition of the STAT3 pathway	[40]
	OVCAR3 cells	Increased cytotoxicity and cell cycle arrest significant proapoptotic and pro-necrotic activities	Modulated BAX/BCL-2 ratio	[41]
Cervical cancer cells	HeLa	Induced apoptotic cell death, inhibited cell proliferation	-	[42]
	C33A, Caski, HeLa cells	Induced apoptosis and inhibit wound healing and migration	Reduced expression of HPV E6 and E7, cyclin A and B, AKT, JNK, p38, and ERK	[43]
Prostate cancer cells	PC-3 cells	Induced apoptotic cell death, inhibited cell proliferation	Decreased expression of Bcl-2, PCA3, upregulated Bax level	[44]
	LNCaP, DU145, PC-3 cells	Inhibited cell growth, induced apoptotic cell death,	Suppression of NF-kB, Bcl-2, Cox-2, increased expression of caspase-3/9	[45]

## Data Availability

Data sharing is not applicable to this article as no new data were created or analyzed in this study.
